# An Approach for the Dynamic Measurement of Ring Gear Strains of Planetary Gearboxes Using Fiber Bragg Gratings

**DOI:** 10.3390/s17122872

**Published:** 2017-12-16

**Authors:** Hang Niu, Xiaodong Zhang, Chenggang Hou

**Affiliations:** 1 School of Mechanical Engineering, Xi’an Jiaotong University, Xi’an 710049, China; nzh1991@stu.xjtu.edu.cn; 2 Key Laboratory of Education Ministry for Modern Design and Rotor-Bearing System, Xi’an Jiaotong University, Xi’an 710049, China; xdzhang@mail.xjtu.edu.cn

**Keywords:** planetary gearbox, ring gear, strain, FBG, dynamic measurement

## Abstract

The strain of the ring gear can reflect the dynamic characteristics of planetary gearboxes directly, which makes it an ideal signal to monitor the health condition of the gearbox. To overcome the disadvantages of traditional methods, a new approach for the dynamic measurement of ring gear strains using fiber Bragg gratings (FBGs) is proposed in this paper. Firstly, the installation of FBGs is determined according to the analysis for the strain distribution of the ring gear. Secondly, the parameters of the FBG are determined in consideration of the accuracy and sensitivity of the measurement as well as the size of the ring gear. The strain measured by the FBG is then simulated under non-uniform strain field conditions. Thirdly, a dynamic measurement system is built and tested. Finally, the strains of the ring gear are measured in a planetary gearbox under normal and faulty conditions. The experimental results showed good agreement with the theoretical results in values, trends, and the fault features can be seen from the time domain of the measured strain signal, which proves that the proposed method is feasible for the measurement of the ring gear strains of planetary gearboxes.

## 1. Introduction

Because of the large transmission ratio and the strong load-bearing capacity, planetary gearboxes are widely used in aero-engines, wind turbines and automobiles [1]. However, due to their heavy load and tough working environments, they are subject to damage of key components [2,3,4]. To prevent shutdown of the entire train and major economic losses, condition monitoring of planetary gearboxes has been attracting considerably increasing attention. At present, vibration signals are mostly used for condition monitoring [5,6]. Although many achievements have been made in this area, the identification of weak faults remains a difficult task. The main reason for this arises from the fact that the vibration signal has a complex relationship with the meshing force of planetary gearboxes. Actually, the meshing force is an ideal signal that can reflect the dynamic behavior of planetary gearboxes, but it’s hard to measure the force. The vibration of planetary gearboxes is induced by gear meshing, so the vibration signal is treated as an alternative of the meshing force. Because vibration sensors are mounted on the housing of the gearbox and there are multiple meshing pairs in a planetary gearbox, vibration signals at different meshing locations have many paths to go from their origins to the sensors through solid mechanical components and their contacts [7]. Vibration signals from different locations have different phases [8], so they can interfere with each other, which may cause the loss of key information. To solve the problem, some researchers have depended on physical models [9,10,11], and others have devoted themselves to studying signal processing methods [1]. These works are all useful, but we think a more fundamental way is to find a new kind of signal that can reflect the meshing force better. The strain of the ring gear is thought to be such a signal [12]. Strain sensors are mounted at the ring gear, which places them closer to the meshing positions. Meanwhile, the strain of the ring gear can change only if the measurement position is near the meshing positon of the planet gear and the ring gear, which makes the signal more stable. For practical applications, the strain of the ring gear must be measured first.

There are mainly two strain measurement methods: the photoelastic method and the electrometric method. The photoelastic method takes advantage of the transient birefringence effect of photoelastic materials in a polarized light field, and the stress optic pattern of the object shows the distribution of stresses (strains). The electrometric method makes use of strain gauges, which are mounted at the measurement area. These two methods both have a long history, and in recent years, they have been used to determine the distribution of tooth root strain [13], to calculate the meshing stiffness under tooth cracks [14,15,16], study the effect of errors and tooth modifications to the tooth root strain [17,18,19], and analyze the contact stress of helical gears under different helical angles [20]. The two traditional methods are very effective in experiments, but they both have some problems to measure the strain of the ring gear in realistic planetary gearboxes with narrow inner space. The photoelastic method requires a complex light system, an open environment and gears made of special materials. Although the strain gauge can have a small size, there can be many wires in the gearbox for distributed measurements, which makes the system complex. Meanwhile, the electrometric method can’t resist the effect of the electromagnetic field.

Fiber Bragg gratings (FBGs) have advantages of small size, high sensitivity and anti-electromagnetic interference. In addition, several FBGs can be made in one optical fiber for distributed measurements, which makes the system simple, so FBGs are considered to be suitable for the real-time measurement of the ring gear strain in realistic planetary gearboxes. Until now, FBGs have been used to monitor the health conditions of many structures, such as ship hulls [21], pipelines [22], geotechnical structures [23] and bridges [24]. However, few studies have been found the strain of the ring gear in planetary gearboxes is measured with FBGs. For the new measurement object, the installation, the sensing characteristics and the measurement system of FBGs have some unique features, which will be analyzed in this paper.

## 2. Primary Principles

### 2.1. FBG

**Figure 1:**
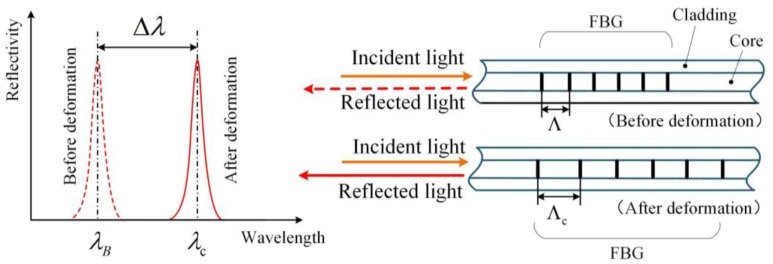
Structure and principle of the FBG.

As shown in Figure 1, the optical fiber consists of the cladding and the core, and the FBG is a special structure in the core. In an optical fiber, the light can be reflected by the FBG, and the wavelength of the reflected light is described as:

(1) $$\lambda_{B}=2n_{eff}\Lambda$$

where Λ is the grating period of the FBG; $n_{eff}$ is the effective refractive index of the core.

When the axial deformation of the FBG occurs (assume that the temperature is constant), the reflected wavelength will change, as shown by:

(2) $$\Delta \lambda =\lambda_{c}-\lambda_{B}=\lambda_{B}(1-p_{e})\varepsilon$$

where $p_{e}$ is the effective elastic optical coefficient, and the typical value is 0.21; $\lambda_{c}$ is the reflected wavelength of the FBG after deformation.

### 2.2. Planetary Gearbox

**Figure 2:**
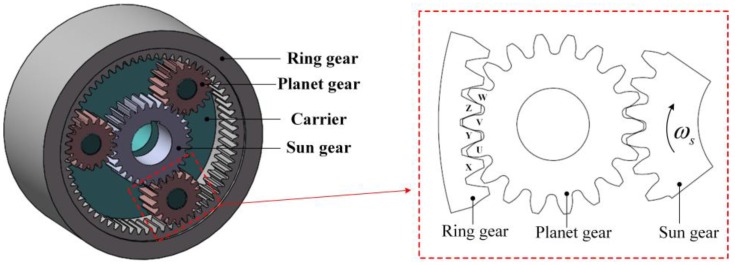
Structure of the planetary gearbox.

As shown in Figure 2, a one-stage planetary gearbox mainly contains a sun gear, few planet gears, a ring gear and a carrier. The ring gear is stationary, the sun gear (input side) and the carrier (output side) can only revolve on its own axis, while the planet gears revolve both round the sun gear and on their own axis. In the planetary gearbox, there are 2*N* meshing pairs (*N* is the number of planet gears). If the load of the planetary gearbox is invariant, the meshing force of each meshing pair is constant, as shown in Equation (3):

(3) $$F_{m}=\frac{T_{L}}{2NC_{sp}\cos\alpha }$$

where $T_{L}$ is the load on the carrier; $C_{sp}$ is the center distance between the sun gear and the planet gear; α is the meshing angle.

However, because the number of tooth pairs involved in a meshing pair changes, the meshing force acting upon a certain tooth in a meshing pair is not constant. As seen in the close view of Figure 2, if we only focus on teeth U, V, W, X, Y and Z, the tooth pairs involved in the ring-planet meshing pair are UX, UX & VY, VY, VY & WZ, and WZ in order, according to the rotation direction of the sun gear. When only one tooth pair is meshed, the meshing force is completely applied to this pair, while if two tooth pairs are meshing, the meshing force will be shared by the two pairs. The sharing principle depends on the meshing stiffness of the two tooth pairs, as shown in Equation (4):

(4) $$\left\{ \begin{array}{l} F_{m,X}=\frac{k_{\mathrm{UX}}}{k_{\mathrm{VY}}\;+\;k_{\mathrm{UX}}}F_{m}\\ F_{m,Y}=\frac{k_{\mathrm{VY}}}{k_{\mathrm{VY}}\;+\;k_{\mathrm{UX}}}F_{m} \end{array}\right.$$

where $F_{m,X}$ is the meshing force acting to the tooth X; $F_{m,Y}$ is the meshing force acting to the tooth Y; $k_{\mathrm{UX}}$ is the meshing stiffness of the tooth pair UX; $k_{\mathrm{VY}}$ is the meshing stiffness of the tooth pair VY. The meshing stiffness can be calculated with the method described in [25].

## 3. Installation of FBGs at the Ring Gear

**Figure 3:**
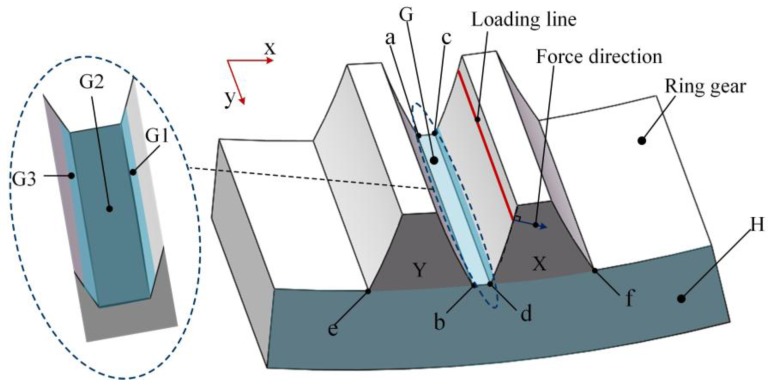
Installation areas of FBGs at the ring gear.

Because of the narrow space inside the planetary gearbox, there are limited positions available on the ring gear for FBGs. Area G (containing surface G1, G2 and G3) and area H in Figure 3 can be two available choices. The length of the two selected areas is enough for FBGs. Meanwhile, FBGs in these areas will not interfere with the meshing motion.

To determine the exact positions of FBGs, the strain distribution of the ring gear is simulated. The parameters of the planetary gearbox used in the simulation are shown in Table 1. The simulation is made by the finite element method (FEM). The simulation model is shown in Figure 3, and the freedoms of the outside surface of the model are constrained. A linear meshing force is applied at the surface of tooth X, and the loading line is parallel to line cd. The problem is solved with a static method. The strain of the ring gear at a moment during the meshing process can be extracted through the post-processing of ANSYS. Other simulations for the strain of the ring gear in this paper are made using the same method, and the differences are the value of the meshing force and the position of the loading line, which are calculated through Equations (3) and (4) according to the moment in the meshing process.

**Figure 4:**
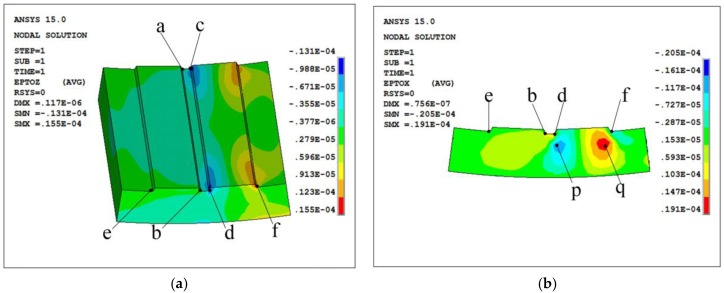
Strain of (**a**) y-direction and (**b**) x-direction.

According to Section 2.1, the FBG is sensitive to its axial deformation, so only the strain of y-direction on area G and the strain of x-direction on area H are focused, as shown in Figure 4. From Figure 4, the maximum strains of area G is at surface G1 near c and d. Meanwhile, the maximum strains of area H is near d, f, p and q. Although the above conclusions are drawn on the premise that tooth X is in meshing, it is obvious that if tooth Y is in meshing, the positions with maximum strains will move to the corresponding positions around tooth Y.

**Figure 5:**
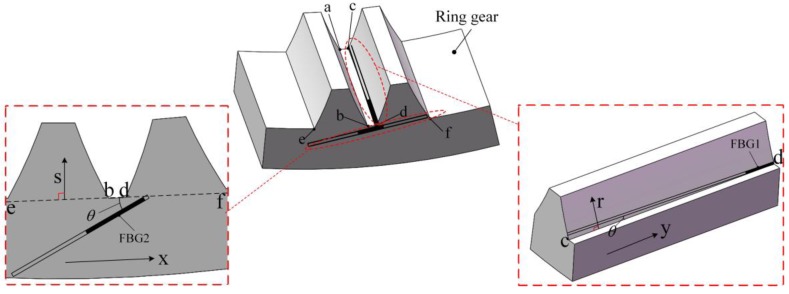
Installation of FBGs.

The FBG should be mounted at the area with the maximum strain (absolute value), as shown in Figure 5. Areas near c and d are equivalent, and FBG1 is just mounted at d for an example. FBG2 isn’t mounted at p or q in Figure 4, because the orientations of the strains at the two points are opposite, and for a ring gear, the distance between p and q is small, the FBG can easily go through the two areas, which makes the FBG have a little deformation and sensitivity. 

Besides the position, the angle of the FBG also need to be considered. Actually the strain of y-direction and x-direction of the selected areas are neither the main components of the strain, which are supposed to be the strain of r-direction and s-direction in Figure 5. Assume that the main component of the strain at the FBG is $\varepsilon_{m}$, then according to the mechanics of materials, the axial strain $\varepsilon_{a}$ of the FBG can be approximatively described as:

(5) $$\varepsilon_{a}=\sqrt{{\left(1+\varepsilon_{m}\right)}^{2}-\frac{{\left(1+\varepsilon_{m}\right)}^{2}-{\left(1+\nu \varepsilon_{m}\right)}^{2}}{1+\tan^{2}\theta }}-1$$

where υ is the Poisson’s ratio of the material of the ring gear; θ is the installation angle.

**Figure 6:**
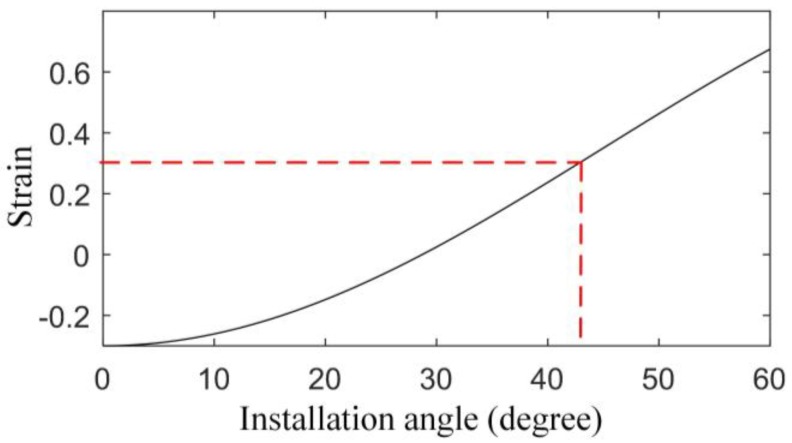
Relationship between the axial strain and the installation angle of the FBG.

According to Equation (5), the relationship between the axial strain of the FBG and the installation angle is expressed in Figure 6 ($\varepsilon_{m}=1$). From Figure 6, the axial strain of the FBG decreases first and then increases as the installation angle increases from 0 degrees. An angle more than 43 degrees can make the strain larger, but the space in the planetary gearbox is too small to provide such an angle, so the installation angle for the FBG is chosen to be 0 degrees, which can make the FBG achieve the maximum sensitivity at the certain position. This installation angle was proved to be reasonable by an experiment discussed in [26].

## 4. Characteristics of the FBG for the Measurement of the Ring Gear Strain

### 4.1. Parameters of the FBG

According to Equation (2), the reflected wavelength of the FBG needs to be obtained to calculate the strain of the ring gear, then the reflected spectrum of the FBG is supposed to have the larger reflectivity, the narrower bandwidth and the higher signal-mode suppression ratio (SMSR) to make the measurement more accurate. For a given optical fiber (usually a single mode fiber), if there is no deformation at the FBG, the grating period only affects the position of the spectrum, but the length of the FBG, the maximum change of the refractive index and the extent of the apodization can all influence the shape of the spectrum. 

**Figure 7:**
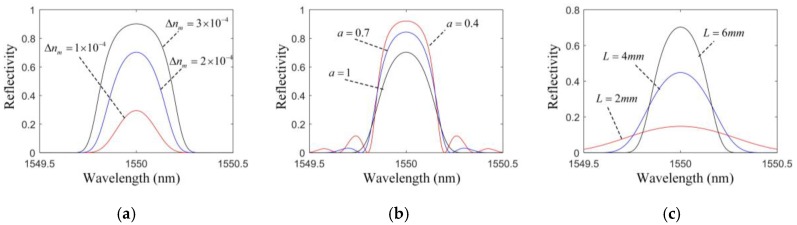
Reflected spectrum of FBGs with different parameters: (**a**) L=6 mm, a=1; (**b**) L=6 mm, $\Delta n_{m}=2\times 10^{-4}$; (**c**) $\Delta n_{m}=2\times 10^{-4}$, a=1.

The transmission matrix method (TMM) is widely used to analyze the reflected light spectrum of fiber gratings [27]. According to this method, the reflected spectrum of FBG with different parameters is simulated in Figure 7. From Figure 7, when the maximum change of the refractive index ($\Delta n_{m}$) decreases, the reflectivity and the bandwidth both decrease. When the extent of the apodization (a) decreases, the reflectivity and SMSR increase, and the bandwidth decrease. This shows $\Delta n_{m}$ and a shouldn’t be too large or too small for measurement accuracy. However, the reflectivity increases and the bandwidth decreases with the increasing length (L), which shows the measurement will be more accurate with a larger length of the FBG. Meanwhile, from Figure 4, the average value along the FBG will become smaller with the increasing length of the FBG, which means that the measuring sensitivity and the signal-to-noise ratio (SNR) will be small.

Based on the above analysis, the maximum reflectivity, the narrowest bandwidth and the highest SMSR can’t be achieved at the same time, so we have to balance these indexes according to the size of the ring gear. The main selected parameters of the FBG for our measurement are shown in Table 2. The original reflected wavelength is 1550 nm, the maximum reflectivity is 0.71, the bandwidth of 3 dB is 0.56 nm, and the SMSR is 10.3 dB.

### 4.2. Simulation of the Measurement

**Figure 8:**
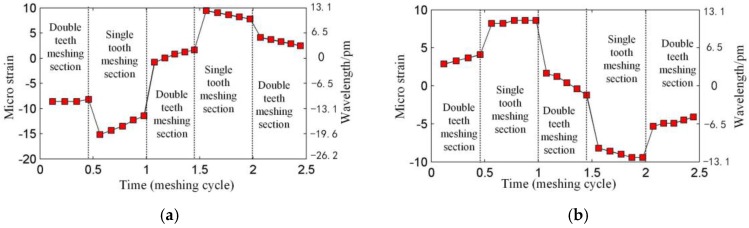
Strains measured by (**a**) FBG1 and (**b**) FBG2.

**Figure 9:**
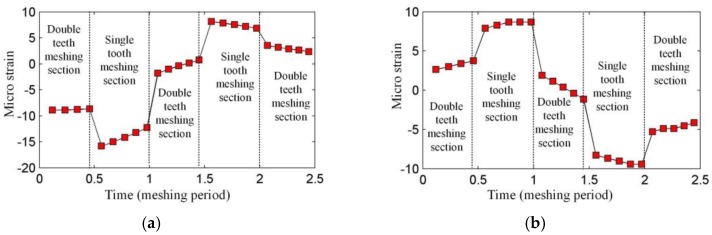
Average strains along (**a**) FBG1 and (**b**) FBG2.

Generally, when the FBG is mounted in an uneven strain field, the reflected spectrum of the FBG will have multiple crests, which makes it impossible to calculate the strain with Equation (2). However, in our application, the strain value and the length of the FBG are small, so there is just one main crest in the reflected spectrum. Although it is possible to obtain the strain along the FBG using optimization algorithms [28], it takes a long time and this not suitable for the dynamic measurement of the ring gear strain. We also want to use Equation (2) to calculate the strain in our application, but the reasonability of this approach needs to be demonstrated, so a simulation of the measurement is made. Firstly, the wavelength shift of the FBG is obtained according to TMM under the strain of the ring gear. Then the measured strain is calculated through Equation (2). The result is shown in Figure 8. From Figure 8, the meshing sections described in Section 2.2 are obvious. Figure 9 shows the average strain of the ring gear along FBG1 and FBG2. The measured strain in Figure 8 and the average strain in Figure 9 are different (This phenomenon is more obvious for the strain concerning FBG1). A reason is that when the FBG is under uneven strain field, the position and shape of the reflected spectrum of the FBG will both change. However, except for the difference, the measurement strain is close to the average one, and they have the same trend, which means the measured strain is valid for our application.

## 5. Measurement System

### 5.1. Principle of the System

**Figure 10:**
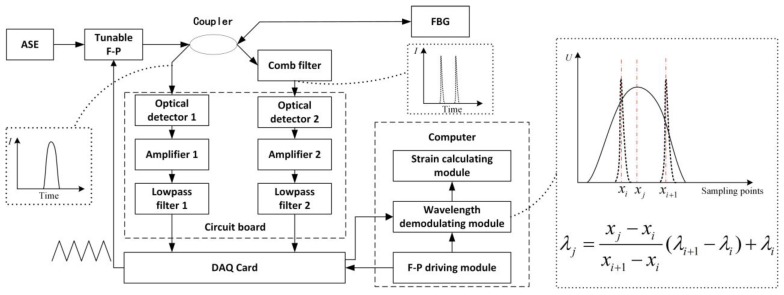
Principle of the measurement system.

The principle of the measurement system is shown in Figure 10. The broadband light from the amplified spontaneous emission (ASE) source enters the tunable Fabry-Perot (F-P) cavity. Only the light with specific wavelengths can pass through the F-P cavity, and the wavelengths depend on the length of the cavity, which is driven by a periodical signal (triangular wave). Actually, the ASE and the tunable F-P cavity are formed to be a scanning light source. The source emits the periodical narrowband light, which has different central wavelengths at different moments in each period. The light is then separated into two parts by the coupler: one goes through the FBG and the other goes through the comb filter (the comb filter is an F-P cavity with a stationary cavity). Then the light spectrums of the two components will appear in the wavelength demodulating module after being processed by the circuit board and collected by the data acquisition (DAQ) card. A close view of the wavelength demodulation process is shown in Figure 10. In the wavelength demodulation module, the crest positions of the spectrums of the FBG ($x_{j}$) and the comb filter ($x_{i}$,$x_{i+1}$) will be found firstly. Because the wavelength of each crest of the comb filter ($\lambda_{i}$,$\lambda_{i+1}$) is known, the wavelength of the FBG ($\lambda_{j}$) can be got by interpolation. Finally, the strain will be calculated through Equation (2).

### 5.2. Paratmeters of the System

#### 5.2.1. Bandwidth

**Figure 11:**
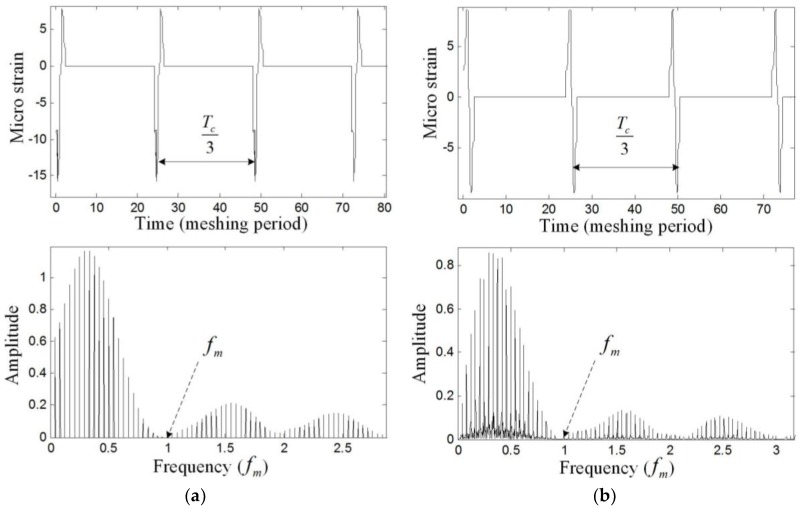
Simulated strain measured by (**a**) FBG1 and (**b**) FBG 2 in the time domain and frequency domain.

The bandwidth of the system is defined as the maximum frequency of the strain that the system can measure. Because the triangular wave is used to drive the tunable F-P cavity, the FBG can be scanned twice in a period, during which two reflected wavelength values of the FBG can be obtained. However, the wavelength demodulating module and the strain calculating module need some time for calculation, so only the upslope of the triangular wave is used to scan the FBG, which means the frequency of the triangular wave is equal to the strain collection frequency. This frequency depends on the frequency of the strain of the ring gear. Figure 11 shows the simulated strain measured by FBG1 and FBG 2 in the time domain and frequency domain. From Figure 11, the measured strain is modulated by the meshing frequency ($f_{m}$) and three times of the carrier frequency ($3f_{c}$). The main frequency components of the strain are within $f_{m}$, so the frequency of the triangular wave ($f_{D}$) is supposed to be at least two times of the meshing frequency ($2f_{m}$) according to the sampling theorem.

#### 5.2.2. Sampling Frequency

**Figure 12:**
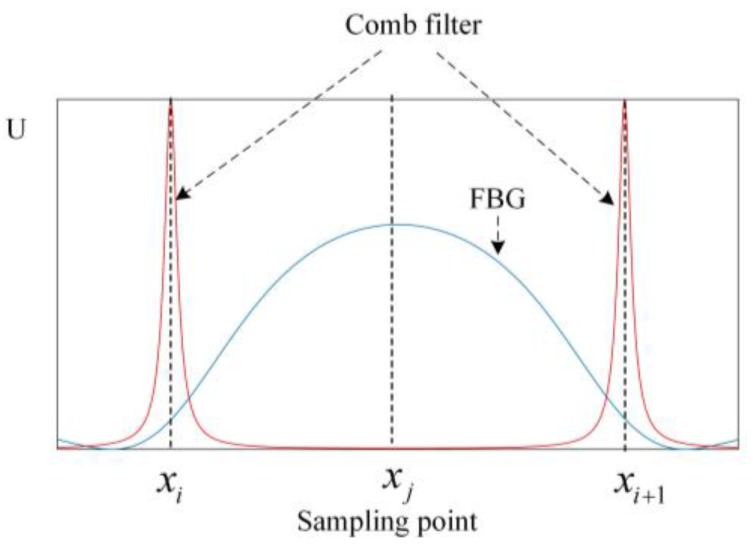
Light spectra of the FBG and the comb filter during the upslope of the triangular wave.

During the upslope of the triangular wave, both the FBG and the comb filter will be scanned once, and the light spectra of the FBG and the comb filter will be obtained, as shown in Figure 12. In Figure 12, the transmitted spectrum of the comb filter is described as:

(6) $$\left\{ \begin{array}{l} \delta_{CF}=\frac{4\pi n_{CF}L_{CF}\cos\theta_{CF}}{\lambda }\\ F_{CF}=\frac{4R_{CF}}{{\left(1\;-\;R_{CF}\right)}^{2}}\\ T_{CF}=\frac{1}{1\;+\;F_{CF}\sin^{2}(\delta_{CF}/2)} \end{array}\right.$$

where $L_{CF}$ is the length of the comb filter; $n_{CF}$ is the refractive index of the comb filter; $\theta_{CF}$ is the angle of incidence; $R_{CF}$ is the reflectivity of the comb filter; $T_{CF}$ describes the transmitted spectrum of the comb filter.

**Figure 13:**
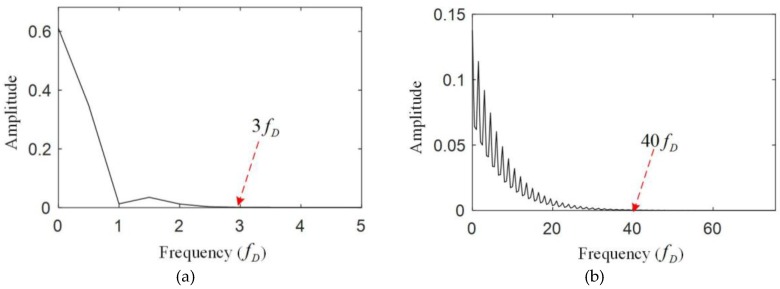
Frequency of the light spectra of (**a**) the FBG and (**b**) the comb filter during the upslope of the triangular wave.

The time for the upslope of the triangular wave is $1/\left(2f_{D}\right)$, and the transmitted spectrum of the comb filter is supposed to be at the central position in the time domain, then the frequency of the light spectra collected by the DAQ card is shown in Figure 13.

From Figure 13, the main frequency components of the FBG spectrum are within $3f_{D}$, and the main frequency components of the comb filter spectrum are within $40f_{D}$, which means the sampling frequency should be at least $80f_{D}$ for the measurement according to the sampling theorem.

#### 5.2.3. Range and Resolving Power

As in Figure 12, we assume that the interval of the two crests in the spectrum of the comb filter is $\lambda_{\mathrm{range}}$, then the range of the measurement system $\varepsilon_{range}$ can be described as $\lambda_{\mathrm{range}}/\left[\lambda_{B}(1-p_{e})\right]$ according to Equation (2). The resolving power of the system $\varepsilon_{resolve}$ can be described as $\varepsilon_{range}/(x_{i+1}-x_{i})$.

### 5.3. Test of the Measurement System

**Figure 14:**
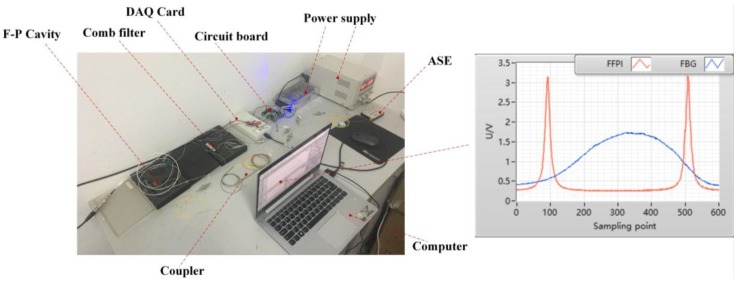
Structure of the measurement system.

The measurement system is established as shown in Figure 14. The tunable F-P cavity is FFT-TF2 (made by MOI, Atlanta, GA, USA). The comb filter is FFP-I (made by MOI, the interval of the adjacent crests is 0.8 nm). DAQ Card is USB 6211 (made by NI, Austin, TX, USA), which has a maximum sampling frequency of 250 kHz (because two input channels are used, the maximum sampling frequency of each channel is 125 kHz). For our application, the maximum input speed of the planetary gearbox is 100 r/min, which means the maximum meshing frequency is 40 Hz (calculated from Table 1). And the maximum load is 30 Nm, which means the range of the strain is less than 30με (Figure 8). According to the theory in Section 5.2, the frequency of the triangular wave is set to be 100 Hz. The range of the triangular wave is set to let the spectrum of the FBG and the comb filter have the form in Figure 12, which makes the measuring range be about 650με. The sampling frequency is set to be 125 kHz, which makes the sampling points between the two crests in the spectrum of the comb filter be about 400 (the close view in Figure 14). Then the resolving power of the system is supposed to be 1.6με.

Because the strain of the cantilever beam can be calculated accurately by the mechanics of materials (Equation (7)), it is used to test the performance of the measurement system:

(7) $$\varepsilon_{c}=\frac{6F_{c}D_{c}}{B_{c}H_{c}{}^{2}E_{c}}$$

where $F_{c}$ is the load at the free end; $D_{c}$ is the distance from the loading position to the measurement position, the value is 134 mm in this experiment; $B_{c}$ is the width of the beam, the value is 30.4 mm; $H_{c}$ is the thickness of the beam, the value is 3.46 mm; $E_{c}$ is the elastic modulus of the steel (C45E4) for the beam, and the value is 210GPa.

**Figure 15:**
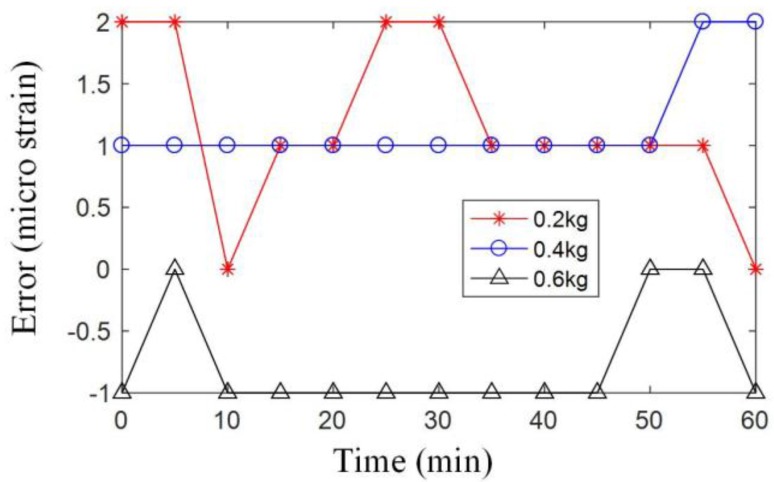
Errors between the measured strain and the theoretical results.

In the experiment, firstly, the FBG is mounted at the specific position of the cantilever beam. Then, different loads (0.2 kg, 0.4 kg, 0.6 kg) are put on the free end of the beam. For each load, the strain is recorded per 5 min for 1 h with the system, meanwhile the strain is calculated by the mechanics of materials. Finally, the error between the measured strain and the theoretical results is analyzed. Table 3 shows the results (the data are rounded to integer) in the experiment. Figure 15 shows the error. From Figure 15, the measurement system has a maximum error of 2με. From the simulation in Figure 8, we know that the maximum tension strain is about 10 micro strain, and the maximum compression strain is about 15 micro strain. The system with 2 micro strain error can capture the main strain values and present the trend of the strain.

## 6. Measurement of the Ring Gear Strain

**Figure 16:**
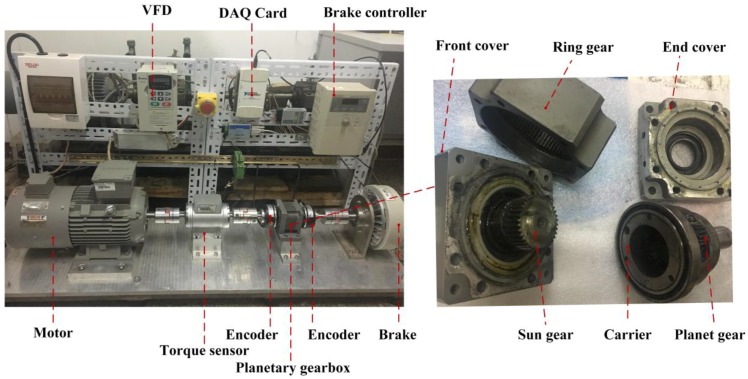
Experimental table.

Figure 16 shows the experimental table. The speed of the planetary gearbox is provided through the motor, which is controlled by the variable-frequency drive (VFD). The load of the planetary gearbox is provided through the brake, which is controlled by the brake controller. The speed and load of the planetary gearbox can be measured by the torque sensor and encoders respectively. The DAQ Card collects the speed and load to the computer, and gives instructions to the VFD and the brake controller. The main parameters of the planetary gearbox have been shown in Table 1. This gearbox is used in the industry, and not specially manufactured for the experiment. 

**Figure 17:**
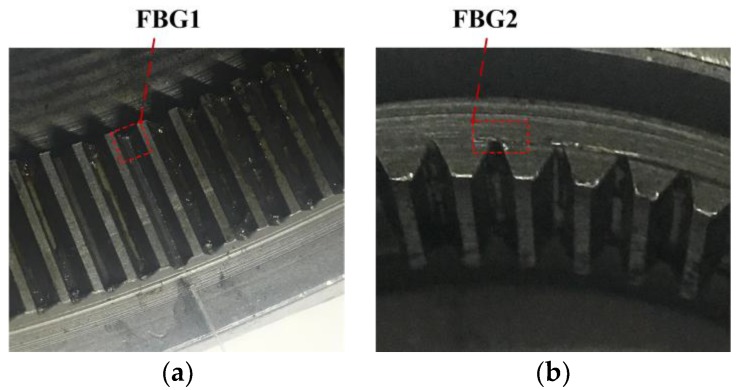
Installation of (**a**) FBG1 and (**b**) FBG2 in the experiment.

**Figure 18:**
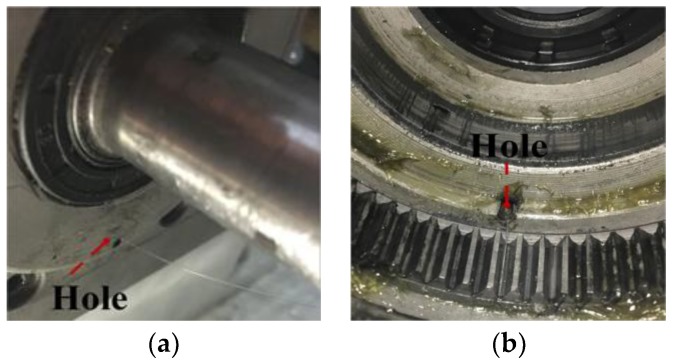
Extraction of the optical fiber: (**a**) a view inside the gearbox; (**b**) a view outside the gearbox.

According to Section 3, the installation of FBG1 and FBG2 is shown in Figure 17. The optical fiber is extracted from the gearbox through a hole in the end cover, as shown in Figure 18.

**Figure 19:**
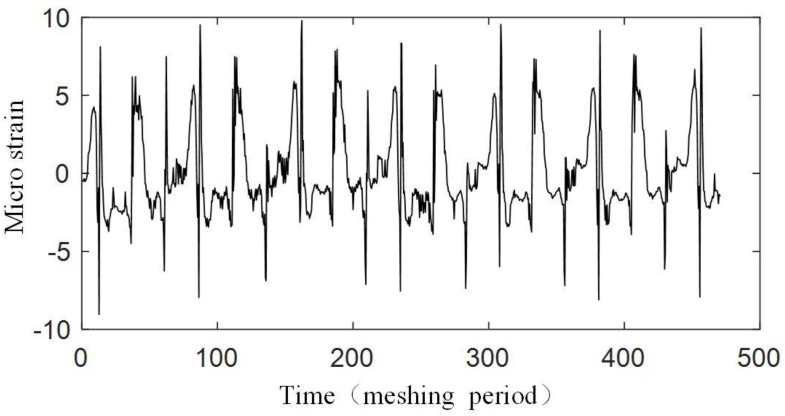
Original strain signal measured by FBG1.

**Figure 20:**
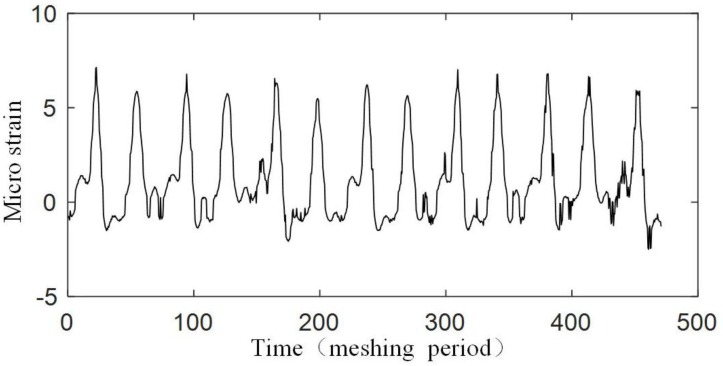
Low-frequency component in the strain signal.

**Figure 21:**
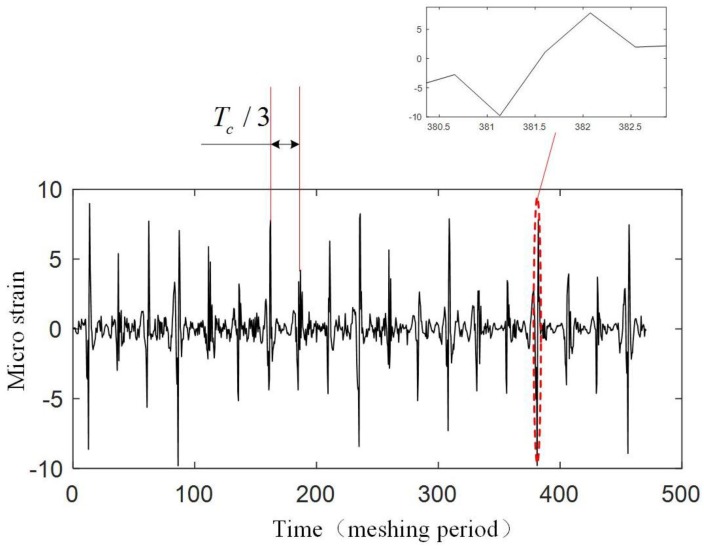
Strain signal measured by FBG1.

**Figure 22:**
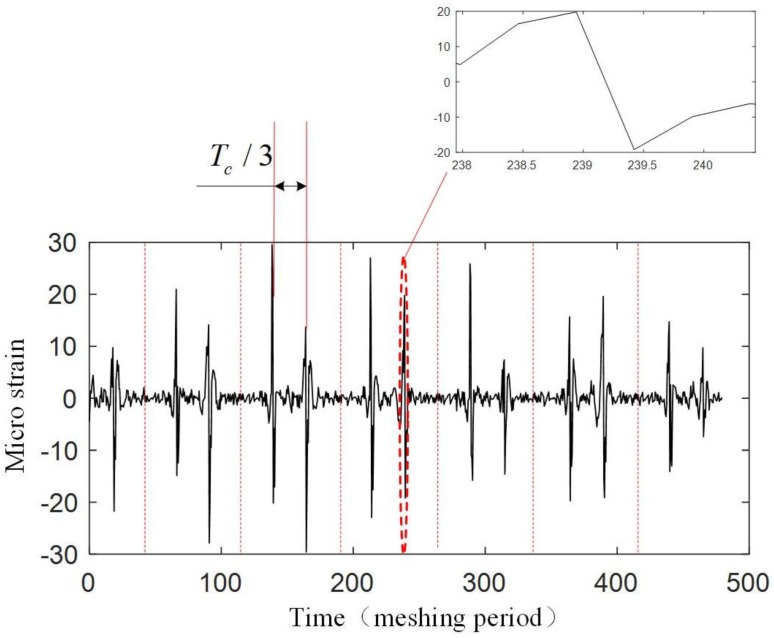
Strain signal measured by FBG2.

The experiment is operated under the condition that the input speed of the planetary gearbox is 100 r/min, and the load of the planetary gearbox is 30 Nm. Figure 19 shows the original strain signal measured by FBG1. The signal mainly contains two components: the pulse-type component and the low-frequency component. In the experiment, when only the bearing of the carrier is installed into the bore of the ring gear and rotates (with no gear meshing), the low-frequency component also appears, but there is no pulse-type component, so it is supposed that the low-frequency component results from the machining error between the bearing of the carrier and the bore of the ring gear. There is interference fits between the bearing and the bore, so when the two structures have errors on coaxiality and circular degree, the ring gear will have deformations during the carrier rotating. Because the frequency of the two components are much more different, the low-frequency component can be removed from the measured signal easily (Figure 20), and the wavelet method is used here. Then if we only consider the effect of the meshing motion, the strain signal measured by FBG1 is shown in Figure 21, and the strain signal measured by FBG2 is shown in Figure 22.

**Figure 23:**
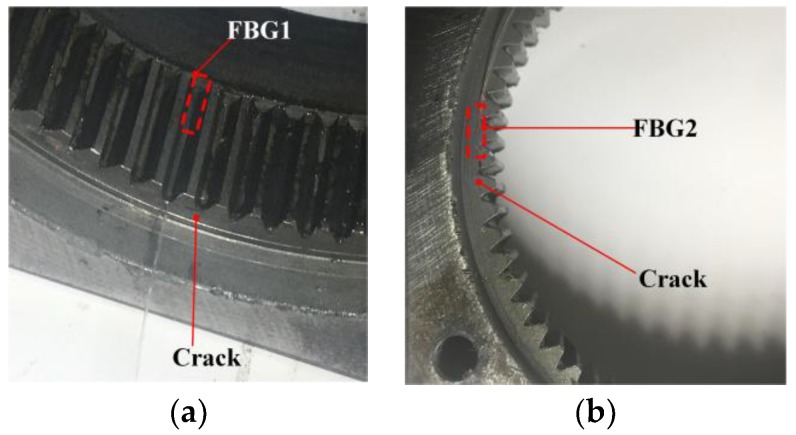
Installation of (**a**) FBG1 and (**b**) FBG2 under a tooth crack.

**Figure 24:**
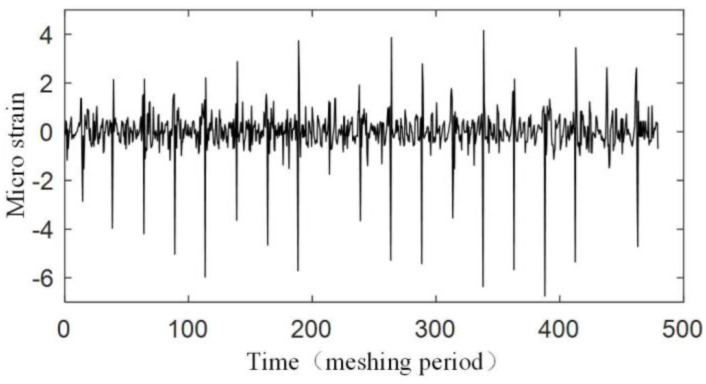
Strain signal measured by FBG1 under a tooth crack.

**Figure 25:**
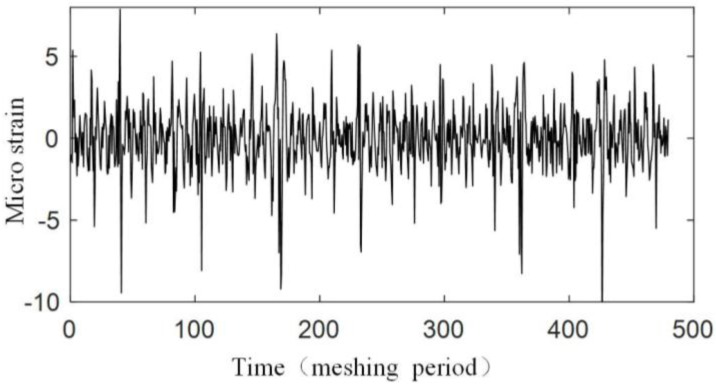
Strain signal measured by FBG2 under a tooth crack.

We also measured the strain signal when there is a crack on the tooth of the ring gear. The installation of FBGs under a tooth crack is shown in Figure 23. The strain signals measured by FBG1 and FBG2 are shown in Figure 24 and Figure 25.

## 7. Discussion

Figure 21 and Figure 22 show that an effective strain signal (caused by the meshing motion) appears every $T_{c}/3$ period just like that shown in Figure 11. The three effective strain signals in the $T_{c}$ period should be completely the same, but in the experiment, they have different values. Especially for the signal measured by FBG2, one effective strain signal almost disappears. The reason is that in the realistic planetary gearbox, the planet gears may have different tooth shapes, different distances to the center of the carrier, and these errors can cause that the planet gears provide different meshing forces to the ring gear.

When focusing on the close views of Figure 21 and Figure 22, we can find that they have the same trends as in Figure 8. However, the middle double meshing section in Figure 8 can hardly be found in Figure 21 and Figure 22. Because the measurement system used in this article have limited bandwidth (100 Hz), some details of the signal will disappear although distortion hasn’t happened according to the sampling theorem. Meanwhile, because the measured strain signal is weak, the error (2με) of the system can also affect the meshing section in the signal. A further observation for the close views shows that the value of the strain measured by FBG1 is closer to the theoretical result in Figure 8, but the value of the strain measured by FBG2 is larger than the theoretical result. The reason is that the strain along FBG2 will have a more complex distribution than that along FBG1, and the measured strain reflects the average level of the strain along FBG (although they are not equal). The maximum strain value along FBG2 is large, but the average value, which depends on the length of FBG2, is smaller. The length of the FBG has been designed to be 2 mm, but it may change because of the machining error of the FBG. A smaller length of the FBG can cause a large strain value.

Only a qualitative analysis for the fault diagnosis experiment is made. The difference can be seen from Figure 21, Figure 22, Figure 24 and Figure 25. The difference is more obvious for the strain measured by FBG2, and it hardly shows the effective strain when there is a tooth crack. For the strain signals measured by FBG1, the value is close, but the distribution of the compression strain and tension strain is much more different. However, for now, the signal is only observed in time domain, more detailed fault features need to be extracted from the time domain, frequency domain and time-frequency domain in further research.

## 8. Conclusions

In this paper, an approach for dynamic measurements of ring gear strains of planetary gearboxes using FBGs is proposed. According to the analysis of the strain distribution of the ring gear, two positions are chosen for the installation of FBGs, and the installation angle of FBGs is determined to be 0°.

Based on the installation, the characteristics of the FBG for the measurement of the ring gear strain is studied. The parameters of the FBG are selected to balance the bandwidth, SMSR and reflectivity of the reflected spectrum according to the size of the ring gear. The strain measured by the FBG is simulated under the uneven strain field, which shows that the measured strain has a close relationship with the average strain along the FBG.

Then a dynamic strain measurement system is built and tested. The bandwidth of the measurement system is 100 Hz, and the accuracy is 2με. Finally, strain measurement experiments in a planetary gearbox are carried out, and the experiment results show good agreement with the theoretical ones. Meanwhile, some features can be seen from the time domain of the measured strain signal when there is a tooth crack at the ring gear.

Based on the achievements in this paper, the distributed measurement research can be carried out with the wavelength division multiplexing (WDM) technology of the FBG in further research, which will finally provide supports for the condition monitoring of planetary gearboxes.

## Figures and Tables

**Table 1:** Parameters of the planetary gearbox.

Parameters	Planet	Sun	Ring
Tooth number	18	36	72
Tooth width/mm	10	10	12
Modification coefficient	0.2664	−0.0103	0.5615
Modulus/mm	0.9		
Meshing angle/degree	20		
Addendum coefficient	1		
Tip clearance coefficient	0.25		
Load/Nm	30		

**Table 2:** Parameters of the FBG.

Λ/μm	L/mm	$\Delta n_{m}$	a
0.5	2	$3.2\times 10^{-4}$	0.1

**Table 3:** Results of the experiment (με).

	Load/kg	0.2	0.4	0.6
Time/min				
**0**		23	41	61
**5**		23	41	62
**10**		21	41	61
**15**		22	41	61
**20**		22	41	61
**25**		23	41	61
**30**		23	41	61
**35**		22	41	61
**40**		22	41	61
**45**		22	41	61
**50**		22	41	62
**55**		22	42	62
**60**		21	42	61
**Theoretical result**		21	41	62
